# Prediction models for infection after kidney transplantation: a systematic review

**DOI:** 10.3389/fmed.2026.1916764

**Published:** 2026-09-10

**Authors:** Xinjian Zhao, Yang Zhang, Haoli Tang, Juan Long, Hao Dong, Gefang Kuang, Jiamei Song

**Affiliations:** Department of Organ Transplantation, Changde Hospital, Xiangya School of Medicine, Central South University (The First people’s Hospital of Changde City), Changde, Hunan, China

**Keywords:** infection, kidney transplantation, machine learning, prediction model, systematic review

## Abstract

**Background:**

Infection after kidney transplantation is a leading cause of graft loss and mortality. Risk prediction models may facilitate early identification of high-risk recipients, yet their quality remains unclear. This study aimed to systematically evaluate the performance, risk of bias, and clinical applicability of prediction models for infection after kidney transplantation. It also sought to provide evidence-based guidance for model selection and future development.

**Methods:**

We searched eight databases from inception to March 2026 for studies developing and validating prediction models for infection after kidney transplantation. Data extraction followed the CHecklist for critical Appraisal and data extraction for systematic Reviews of prediction Modelling Studies (CHARMS), and the Prediction model Risk Of Bias Assessment Tool (PROBAST) was used to assess risk of bias and applicability.

**Results:**

15 studies involving 20 models were included. All studies were retrospective and covered urinary tract infection, pulmonary infection, bloodstream infection (BSI), BK virus activation, and overall infection. The internally validated area under the curve (AUC) ranged from 0.63 to 0.925, with nine studies reporting an AUC above 0.80. However, these promising internal estimates should be interpreted with caution, as they may be inflated by optimism bias given the uniformly high risk of bias identified across all included studies. Only three studies undertook external validation. The PROBAST assessment revealed a high overall risk of bias in all studies. Frequently identified predictors were albumin, age, diabetes, kidney function, and donor type.

**Conclusion:**

Current models show acceptable discrimination internally but have high risk of bias from methodological flaws. External validation and calibration assessment are insufficient. Future prospective multicenter studies should standardize predictor selection and missing-data handling while ensuring rigorous external validation.

**Systematic review registration:**

https://www.crd.york.ac.uk/prospero/, identifier CRD420261336728.

## 1 Introduction

Kidney transplantation is the most effective treatment for end-stage renal disease (ESRD) (1). Patient survival has improved markedly, driven by advances in surgical techniques and the refined immunosuppressive regimens. Nevertheless, although immunosuppression effectively prevents graft rejection, it concurrently impairs the host immune defense against pathogens. This immunosuppressed state places recipients at sustained risk of opportunistic infections (2). Up to 74% of recipients experience at least one urinary tract infection (UTI) within the first year after kidney transplantation (3). Cytomegalovirus (CMV) reactivation occurs in 50–90% of patients. BK polyomavirus (BKPyV) DNAemia develops in approximately 30% of cases, with 5%–10% progressing to BKPyV-associated nephropathy (4, 5). Pulmonary infection affects 8.3%–63.6% of recipients (6), and bloodstream infection (BSI) occurs in approximately 11.0% (7).

Infection-related complications substantially increase the risk of graft loss and remain a leading cause of mortality in kidney transplant recipients. Bocchi et al. (8) examined the association between infection and outcomes in a cohort of 1,399 living-donor kidney transplant recipients. Patients who developed two or more significant infections within 6 months after kidney transplantation had a markedly higher risk of graft loss. A meta-analysis further reported that infected recipients had a 10.26-fold higher overall mortality risk than those who remained without infection. In that meta-analysis, recipients who received antithymocyte globulin (ATG) induction therapy faced an even higher post-infection mortality risk. Corresponding hazard ratios were 6.23 for pulmonary infection and 5.56 for BSI (9). Infection-related complications have thus emerged as a major clinical challenge in transplantation medicine. Balancing immunosuppression against infection prevention and accurately identifying individuals at high risk for targeted prevention, has become urgent clinical needs. A risk quantification approach integrating multidimensional patient characteristics can define the target population more precisely than empirical antimicrobial prophylaxis. This approach could also reduce the harms associated with indiscriminate antimicrobial use. This unmet clinical need has drawn increasing attention to the development of prediction models.

Risk stratified, individualized infection management has been adopted as a core strategy by multiple authoritative international and national guidelines. The International CMV Consensus Guidelines recommend risk stratification based on donor and recipient CMV serostatus, and advocate CMV-specific cell-mediated immunity assays to guide de-escalation of prevention strategies (10). The International BK Virus Consensus Guidelines recommend risk stratified viral load screening protocols based on well-defined risk factors (11). The Chinese Clinical Practice Guideline on Immune Monitoring in Kidney Transplantation advocates early detection of infection risk through active immune monitoring, and the use of this information to guide individualized immunosuppression adjustment (12). Prediction models are statistical tools that integrate multiple variables to estimate an individual’s risk of a specific outcome. A growing number of studies have developed and validated prediction models for infection after kidney transplantation (5, 13–15). These models typically incorporate recipient immune status, donor characteristics, perioperative exposures, and microbiological markers. Such integration enables stratified risk assessment, which can guide timely antimicrobial prophylaxis, dynamic immunosuppression adjustment, and optimized monitoring after transplantation. Despite these advances, existing models show considerable heterogeneity in predictive performance, clinical applicability, and external validity. Moreover, no systematic review has yet comprehensively synthesized prediction models for infection after kidney transplantation.

This systematic review aims to synthesize the existing evidence on prediction models for infection after kidney transplantation. It also seeks to evaluate the predictive performance of these models and to offer methodological recommendations for developing clinically translatable prediction tools.

## 2 Methods

The research question was defined according to the PICOTS framework (Supplementary Table 1). The protocol was prospectively registered with PROSPERO (CRD420261336728). This systematic review followed the Preferred Reporting Items for Systematic Reviews and Meta-Analyses (PRISMA) guidelines (16). Data extraction was guided by the Checklist for critical Appraisal and data extraction for systematic Reviews of prediction Modelling Studies (CHARMS) (17).

### 2.1 Literature search strategy

This study systematically searched eight databases, including Web of Science, the Cochrane Library, PubMed, Embase, SinoMed, VIP (China Science and Technology Journal Database), CNKI, and Wanfang Data. The search covered records from database inception to March 26, 2026. The search strategy combined subject headings and free-text terms for the core concepts “kidney transplantation,” “infection,” and “prediction model,” adapted for each database. The full search strategy is available in Supplementary Table 2.

### 2.2 Eligibility criteria

The inclusion criteria were as follows. (1) The study population comprised patients who had undergone kidney transplantation. (2) The study developed and validated a risk prediction model for infection after kidney transplantation. (3) The model included at least two predictors. (4) The study design was observational, including prospective cohort studies, retrospective cohort studies, or case-control studies. (5) The study reported performance metrics, including the area under the receiver operating characteristic curve (AUROC), sensitivity, and specificity.

The exclusion criteria were as follows. (1) Studies developing models exclusively in pediatric kidney transplant recipients. (2) Studies that built a model without any validation, as this precludes assessment of overfitting or generalizability to new datasets. (3) Studies with unavailable full text or substantial missing key data. (4) Duplicate publications. (5) Review articles, animal studies, conference abstracts, and cross-sectional studies.

### 2.3 Literature screening and data extraction

Two researchers independently performed literature screening and data extraction. Retrieved records were managed in EndNote X9 for duplicate removal. The researchers screened the titles and abstracts to exclude studies that clearly did not meet the inclusion criteria. Full texts of the remaining studies were read for final inclusion decisions. Disagreements were resolved by discussion, with adjudication by a third senior researcher when necessary. Data extraction was performed using a standardized form designed according to the CHARMS checklist (17). Different algorithms or separate modeling approaches for the same outcome were treated as distinct models. Information extracted included first author, publication year, country, study design, sample size, predictor selection method, modeling method, validation strategy, discrimination metrics, calibration method, and missing data handling. The extracted data were recorded into an Excel spreadsheet for collation and summarization.

### 2.4 Quality assessment and risk of bias evaluation

Two researchers independently assessed risk of bias and applicability of the included studies using the Prediction model Risk of Bias Assessment Tool (PROBAST) (18). The tool evaluates four domains including participants, predictors, outcome, and analysis comprising 20 signaling questions. Each signaling question is answered as “yes/probably yes,” “no/probably no,” and “no information.” Based on the responses, each domain’s risk of bias was rated as “low,” “high,” or “unclear.” Overall risk of bias was judged low when all four domains were rated low, and high when any domain was rated high. Applicability was assessed with the same three dimensions (participants, predictors, and outcome) using criteria analogous to those for risk of bias.

## 3 Results

### 3.1 Literature screening process and results

**FIGURE 1:**
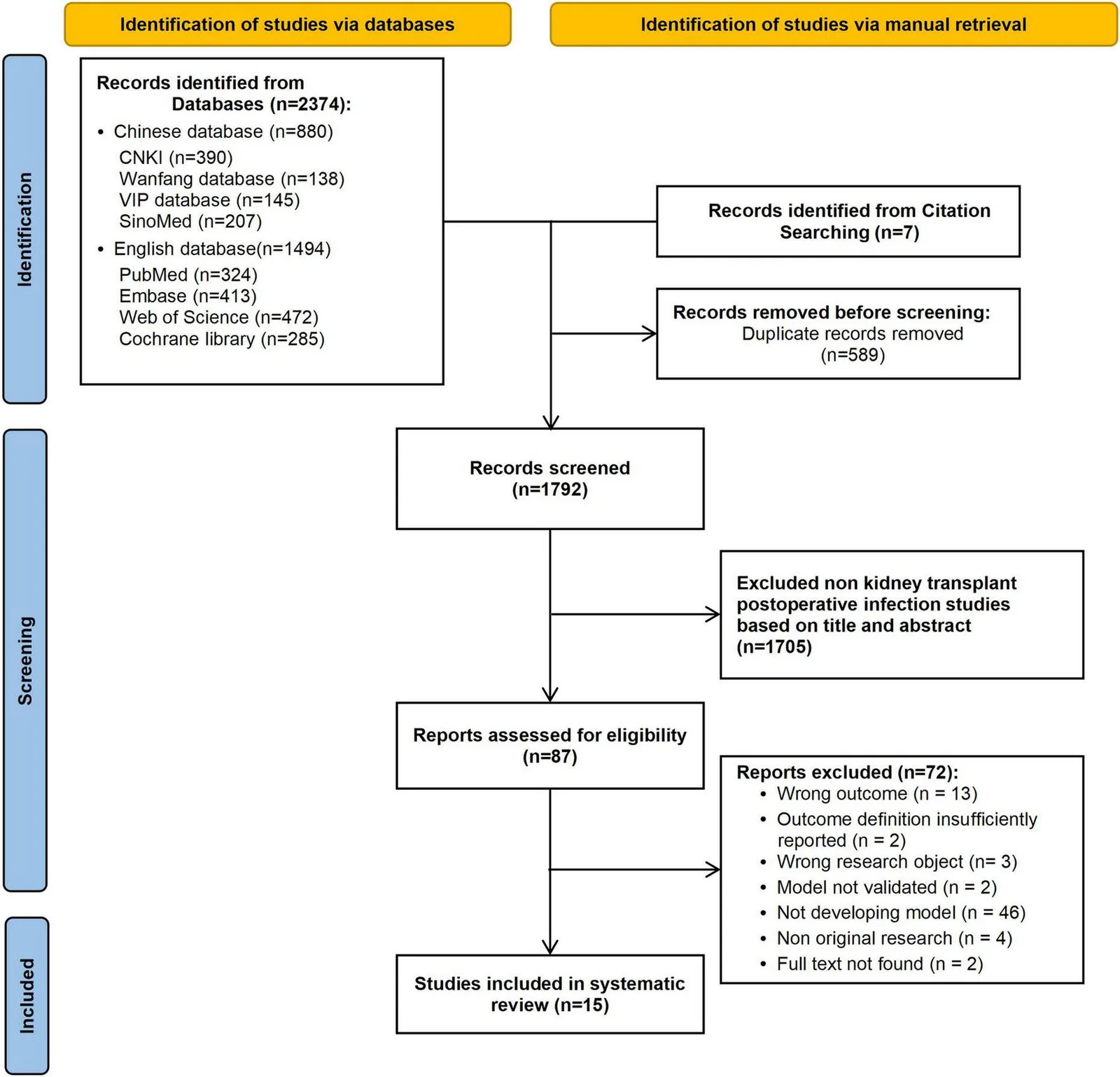
Preferred Reporting Items for Systematic Reviews and Meta-Analyses (PRISMA) flow diagram of the literature screening process.

This study combined a systematic database search with a manual search. Database searches yielded 2,374 records, and citation tracking plus manual searching identified 7 additional records. After deduplication, 1,792 records remained. Title and abstract screening excluded 1,705 records that were unrelated to the topic or did not focus on infection after kidney transplantation. The remaining 87 records underwent full-text review. During this review, 72 additional records were excluded according to the prespecified eligibility criteria. Ultimately, 15 studies on prediction models for infection after kidney transplantation were eligible and included. Figure 1 (PRISMA flowchart) illustrates the complete screening process.

### 3.2 Basic characteristics of the included studies

A total of 15 studies on prediction models for infection after kidney transplantation were included (5–7, 13–15, 19–27), with publication years ranging from 2020 to 2026. Thirteen studies were conducted in China (6, 7, 13–15, 19–24, 26, 27), one in Spain (25), and one in the United States (5). All studies were retrospective. The sample sizes ranged from 77 to 932 participants. The largest and only multicenter study, by Fernández-Ruiz et al., included 932 participants (25). All studies focused on kidney transplant recipients. 12 explicitly included adult recipients (5–7, 13–15, 19, 22–26). One study explicitly excluded pediatric recipients (27). The rest did not specify age restrictions. Three explicitly included first-transplant recipients (6, 20, 21).

The outcomes covered common types of infection after kidney transplantation. Five focused on pulmonary infection (6, 13, 20, 21, 27), four on overall infection (14, 15, 24, 25), three on BK virus infection (5, 23, 26), two on UTI (19, 22), and one on BSI (7). All provided clear diagnostic criteria or clinical definitions for their outcomes. The incidence of infection varied from 8.3% to 63.6% across studies. Specifically, BK virus activation incidence ranged from 13.4% to 60.5% (5, 23, 26), pulmonary infection ranged from 8.3% to 63.6% (6, 13, 20, 21, 27), and UTI ranged from 26.4% to 40.3% (19, 22). Most defined the observation window as the first year post-transplant (5, 6, 15, 19–22). Others set it within 6 months (7), within 30 days (14), the hospitalization period (27), or between 1 and 6 months post-transplant (25). The remaining studies defined outcomes by routine follow-up (23, 26) or reported median time to infection (24). Ten studies clearly reported donor sources including donation after brain death, donation after circulatory death, and living donors (5, 13–15, 19, 21–23, 25, 27). The rest did not detail donor type. Supplementary Table 3 presents the main characteristics of the included studies.

### 3.3 Characteristics and performance of prediction models

#### 3.3.1 Predictor selection methods

Nine studies used univariate analysis combined with multivariable logistic regression to select predictors (5, 6, 13, 14, 19, 21–23, 25). Six studies introduced shrinkage methods or machine learning approaches, such as the Least Absolute Shrinkage and Selection Operator (LASSO) regression and recursive feature elimination, to control the risk of overfitting (7, 15, 20, 24, 26, 27).

#### 3.3.2 Number and types of predictors

The number of predictors ranged from 3 to 18 across the included studies. The predictors could be summarized into four broad categories. (1) Demographic and clinical characteristics, including age, sex, smoking history, diabetes history, and hypertension history. (2) Laboratory indicators, such as albumin, serum uric acid, platelet count, neutrophil count, lymphocyte count, estimated glomerular filtration rate, and procalcitonin. (3) Surgery- and transplant-related factors, including donor type, operation duration, catheter indwelling time, acute rejection, delayed graft function, and induction therapy regimen. (4) Radiomics features, which were reported in only one study (20). Among all predictors, albumin (7, 14, 15, 21, 24, 26, 27), age (5, 6, 15, 25–27), and diabetes or hyperglycemia (7, 15, 21, 22) were the most frequently included.

#### 3.3.3 Modeling methods

Logistic regression was used as the modeling method in 13 studies (5–7, 13, 15, 19–25, 27). One study built five types of models, including logistic regression, support vector machine, random forest, naive Bayes, and adaptive boosting (AdaBoost) (27). Another study used static and dynamic Cox regression model (26).

#### 3.3.4 Handling of continuous variables and missing data

Four studies categorized continuous variables (5, 13, 21, 25), while the other studies used continuous data directly. Seven studies directly excluded cases with missing data (6, 13, 14, 19–21, 27). Five studies did not report how missing data were handled (5, 15, 23, 24, 26). Only three studies clearly reported how missing data were handled. Two studies performed multiple imputation for variables with a missing rate below a certain threshold (7, 22). One study used complete-case analysis in the primary analysis and multiple imputation in the sensitivity analysis (25). The remaining studies did not clearly describe the handling of missing data.

#### 3.3.5 Model validation methods

Thirteen studies reported internal validation methods. The main methods included random split (14, 22, 24), cross-validation (6, 26), and bootstrap resampling (13). Some studies combined multiple internal validation methods to enhance the robustness of the results (5, 7, 15, 19, 20, 25, 27). Only three studies performed external validation (21, 23, 25).

#### 3.3.6 Model calibration and presentation format

Twelve studies reported model calibration methods, mainly the Hosmer-Lemeshow test (7, 13–15, 20–23, 25) and calibration plots (5, 7, 13–15, 19, 22, 24, 25). The remaining three studies did not report calibration (6, 26, 27). Eight studies presented the model as a nomogram (7, 13–15, 19, 22–24), two as an integer risk score (5, 25), two as a logistic regression equation (6, 21), one as a dynamic Cox regression equation (26), and two studies did not clearly report the presentation format (20, 27).

#### 3.3.7 Model discrimination and predictive performance

15 studies involving 20 models. Thirteen studies (5–7, 13–15, 19, 20, 22, 24–27) reported the internally validated area under the curve (AUC) values, which ranged from 0.63 to 0.925. Among them, nine studies (6, 7, 14, 15, 19, 20, 22, 24, 27) achieved an AUC above 0.80, indicating good discriminative ability. The model for BSI showed the highest AUC of 0.925 (95% CI: 0.884–0.955) (7). The prediction model for early infection after kidney transplantation had an AUC of 0.914 (95% CI: 0.861–0.967) (14). The model for overall infection had an AUC of 0.900 (95% CI: 0.860–0.940) (15). The model for pulmonary infection had an AUC of 0.897 (6). The model for UTI had an AUC of 0.841 (95% CI: 0.619–1.00) (19). One study on predicting severe pneumonia after kidney transplantation compared the predictive performance of five machine learning algorithms, and the random forest model performed best (AUC = 0.91) (27). Another study used a dynamic Cox regression model to predict BK virus activation after kidney transplantation. The static Cox model had an AUC of 0.63, while the dynamic model achieved an AUC of 0.70 ± 0.03 (26).

Only one study reported both internal and external validation (25). This study predicted overall infection after kidney transplantation, with an internally validated AUC of 0.744 (95% CI, 0.689–0.793) and an externally validated AUC of 0.730 (95% CI, 0.666–0.794). In the bacterial infection subgroup, the internal validation AUC was 0.767 (95% CI, 0.706–0.827) and the external validation AUC was 0.734 (95% CI, 0.673–0.794). These results indicate a slight decline in discrimination from internal to external validation, though the performance remained at an acceptable level. Two other studies (21, 23) reported only external validation results. The external validation AUC was 0.834 (95% CI, 0.661–0.978) for the pulmonary infection model (21) and 0.699 (95% CI, 0.590–0.808) for the BK virus activation model (23). Eight studies (7, 13, 14, 19, 20, 22, 24, 27) reported sensitivity and specificity. Sensitivity ranged from 0.08 to 0.962, and specificity ranged from 0.362 to 0.99. Four studies (13, 20, 24, 26) reported model accuracy, which ranged from 0.65 to 0.788. The characteristics and performance of the prediction models are summarized in Table 1 and Supplementary Table 4.

**TABLE 1:** Performance of the prediction models in the included studies.

Study	AUC (95% CI)	Accuracy	Sensitivity	Specificity
Chu and Ren (19)	0.841 (0.619–1.00)		0.857	0.889
Guo et al. (20)	0.85 (0.718–0.982)	0.788	0.7	0.923
Yao et al. (6)	0.897			
Gong et al. (21)	External validation: 0.834 (0.661–0.978)			
Cui et al. (7)	0.925 (0.884–0.955)		0.962	0.872
Zhang et al. (15)	0.900 (0.860–0.940)			
Bian (22)	0.878 (0.832–0.923)		0.738	0.834
Wang et al. (23)	External validation:0.699 (0.590–0.808)			
Chen et al. (24)	0.81	0.77	0.81	0.74
Fernández-Ruiz et al. (25)	Internal: Overall infection 0.744 (0.689–0.793) Bacterial infection 0.767 (0.706–0.827) External: Overall infection 0.730 (0.666–0.794) Bacterial infection 0.734 (0.673–0.794)			
Pan et al. (13)	0.705 (0.631–0.799)	0.778	0. 919	0.362
Yamauchi et al. (5)	0.65 (0.54–0.77)			
Fang et al. (26)	Static Cox model 0.63 Dynamic model 0.70 ± 0.03	Static model 0.65 Dynamic model 0.76		
Luo et al. (27)*	Random Forest 0.91, LR 0.86, SVM 0.82, AdaBoost 0.84, Naive Bayes 0.65		Random Forest 0.67 LR 0.46 SVM 0.21 AdaBoost 0.54 Naive Bayes 0.08	Random Forest 0.97 LR 0.94 SVM 0.98 AdaBoost 0.92 Naive Bayes 0.99
Xu et al. (14)	0.914 (0.861–0.967)		0.903	0.832

LR, logistic regression; RF, random forest; SVM, support vector machine; AdaBoost, adaptive boosting. *Metrics derive from an oversampled test set.

### 3.4 Risk of bias assessment and applicability

The overall risk of bias was high for all 15 included studies (5–7, 13–15, 19–27). In the participants domain, all studies were rated high risk due to their retrospective design and reliance on electronic medical records or clinical databases, sources that may introduce selection and recall bias. In the predictors and outcomes domains, only one study was rated low risk (25). The remaining 14 studies did not report whether blinding was used during predictor assessment and outcome determination, and were therefore rated as unclear. In the analysis domain, all studies were rated as high risk. Among them, 9 studies (6, 7, 15, 19–21, 23, 26, 27) had insufficient sample sizes, with an events per variable (EPV) below 20. A study can be rated as “Yes” despite an EPV of only 19, owing to external validation with 105 events and the stability demonstrated by bootstrap correction (25). Nine studies selected predictors based on *P*-values from univariable analysis (5, 6, 13, 14, 19, 21–23, 25, 26). Seven studies directly excluded participants with missing data (6, 13, 14, 19–21, 27), whereas five studies did not clearly report their handling of missing data (5, 15, 23, 24, 26). Three studies (6, 26, 27) did not perform model calibration assessment. Three studies (14, 22, 24) used only random data splitting for internal validation. Regarding applicability, 11 studies were rated as low concern (5, 7, 13–15, 19, 22–25, 27). The remaining 4 studies had a high risk of applicability concerns in the participant domain, because they excluded patients with prior kidney transplants (6, 20, 21, 26). The PROBAST signaling question level table and detailed results of the risk of bias assessment are provided in Tables 2, 3.

**TABLE 2:** Prediction model risk of bias assessment tool (PROBAST) signaling question level table.

PROBAST domain and signaling questions	Yes/probably yes	No/probably no	No information
Domain 1: participants			
1.1 Were appropriate data sources used, e.g., cohort, randomized controlled trial, or nested case–control study data?	0	15 (100%)	0
1.2 Were all inclusions and exclusions of participants appropriate?	15 (100%)	0	0
Domain 2: predictors			
2.1 Were predictors defined and assessed in a similar way for all participants?	15 (100%)	0	0
2.2 Were predictor assessments made without knowledge of outcome data?	1 (6.7%)	0	14 (93.3%)
2.3 Are all predictors available at the time the model is intended to be used?	15 (100%)	0	0
Domain 3: outcome			
3.1 Was the outcome determined appropriately?	15 (100%)	0	0
3.2 Was a pre-specified or standard outcome definition used?	15 (100%)	0	0
3.3 Were predictors excluded from the outcome definition?	15 (100%)	0	0
3.4 Was the outcome defined and determined in a similar way for all participants?	15 (100%)	0	0
3.5 Was the outcome determined without knowledge of predictor information?	1 (6.7%)	0	14 (93.3%)
3.6 Was the time interval between predictor assessment and outcome determination appropriate?	15 (100%)	0	0
Domain 4: analysis			
4.1 Were there a reasonable number of participants with the outcome?	6 (40%)	9 (60.0%)	0
4.2 Were continuous and categorical predictors handled appropriately?	11 (73.3%)	4 (26.7%)	0
4.3 Were all enrolled participants included in the analysis?	15 (100%)	0	0
4.4 Were participants with missing data handled appropriately?	3 (20.0%)	12 (80.0%)	0
4.5 Was selection of predictors based on univariable analysis avoided?	6 (40.0%)	9 (60.0%)	0
4.6 Were complexities in the data accounted for appropriately?	15 (100%)	0	0
4.7 Were relevant model performance measures evaluated appropriately?	12 (80%)	3 (20.0%)	0
4.8 Was model overfitting, underfitting and optimism in model performance accounted for?	12 (80%)	3 (20.0%)	0
4.9 Do predictors and their assigned weights in the final model correspond to the results from the reported multivariable analysis?	13 (86.7)	2 (13.3%)	0

**TABLE 3:** The results of the assessment of included studies.

Study	ROB				Applicability			Overall	
	Participants	Predictors	Outcomes	Analysis	Participants	Predictors	Outcomes	ROB	Applicability
Chu and Ren (19)	High	Unclear	Unclear	High	Low	Low	Low	High	Low
Guo et al. (20)	High	Unclear	Unclear	High	High	Low	Low	High	High
Yao et al. (6)	High	Unclear	Unclear	High	High	Low	Low	High	High
Gong et al. (21)	High	Unclear	Unclear	High	High	Low	Low	High	High
Cui et al. (7)	High	Unclear	Unclear	High	Low	Low	Low	High	Low
Zhang et al. (15)	High	Unclear	Unclear	High	Low	Low	Low	High	Low
Bian (22)	High	Unclear	Unclear	High	Low	Low	Low	High	Low
Wang et al. (23)	High	Unclear	Unclear	High	Low	Low	Low	High	Low
Chen et al. (24)	High	Unclear	Unclear	High	Low	Low	Low	High	Low
Fernández-Ruiz et al. (25)	High	Low	Low	High	Low	Low	Low	High	Low
Pan et al. (13)	High	Unclear	Unclear	High	Low	Low	Low	High	Low
Yamauchi et al. (5)	High	Unclear	Unclear	High	Low	Low	Low	High	Low
Fang et al. (26)	High	Unclear	Unclear	High	High	Low	Low	High	High
Luo et al. (27)	High	Unclear	Unclear	High	Low	Low	Low	High	Low
Xu et al. (14)	High	Unclear	Unclear	High	Low	Low	Low	High	Low

Rob, risk of bias; low, low ROB/low concern regarding applicability; high, high ROB/high concern regarding applicability; unclear, unclear ROB/unclear concern regarding applicability.

## 4 Discussion

### 4.1 Main findings

Infection after kidney transplantation is common and strongly associated with prolonged hospital stay, graft dysfunction, and increased mortality (8). Risk prediction models can identify high-risk individuals preemptively, thereby enabling early intervention. This systematic review identified 15 eligible studies on prediction models for infection after kidney transplantation. The number of predictors per model ranged from 3 to 18. Frequently identified predictors across infection types included albumin, age, diabetes or glycemia, kidney function indicators, and donor source. Internally, most models showed good discriminative ability (AUC > 0.80) and appeared to effectively identify patients at high risk of infection after kidney transplantation. Nevertheless, the PROBAST assessment revealed a uniformly high risk of bias across all included studies. Notable deficiencies were identified in external validation, calibration assessment, and missing data handling. These limitations constrain the clinical applicability of existing models and highlight the need for further high-quality studies.

### 4.2 Heterogeneity in infection incidence

The reported incidence of infection among the included studies varied substantially, ranging from 8.3% to 63.6%. This wide variation likely reflects multiple underlying drivers. The choice of immunosuppressive regimen is one such factor. Induction therapy with lymphocyte depleting agents, particularly rATG, has been consistently associated with higher infection risk compared with basiliximab (28). In the maintenance phase, cyclosporine-based protocols have been linked to a greater burden of severe infections relative to tacrolimus-based regimens (29), whereas mTOR inhibitor-based strategies appear to confer a protective effect against CMV infection (30). Antimicrobial prophylaxis practices are far from standardized. A nationwide survey conducted in China documented up to 10 distinct perioperative antibiotic regimens across transplant centers (31). Follow-up duration also plays a critical role. Data from a large United States Renal Data System cohort of 141,661 kidney transplant recipients show that the cumulative infection rate climbs from 36.9% at 3 months to 53.7% at 1 year and 78.0% at 5 years (32). Consistent with this trajectory, the studies in our review employed widely differing observation windows, ranging from 30 days to routine clinical follow-up. This variability in follow-up inevitably drives variation in the reported cumulative incidence. Heterogeneity in outcome definitions further complicates cross-study comparisons. Some studies focused on site- or pathogen-specific infections, whereas others adopted composite endpoints of overall infection, yielding disparate estimates. Study populations also differed between studies. Some trials were restricted to first-time recipients, while others included retransplant cases. Notably, allograft source remains a relevant factor. Recipients of deceased donor kidneys face a higher infection burden than those receiving living donor grafts (33). Geographical representation was uneven as well. The majority of studies originated from China (*n* = 13), with only one each from Spain and the United States. Regional differences in endemic pathogen profiles and clinical management practices may therefore contribute additional variability to the observed infection rates (34).

### 4.3 Model performance and risk of bias

This systematic review identified 15 studies reporting 20 prediction models for infection after kidney transplantation. These models addressed several outcome types, including pulmonary infection, overall infection, BK virus reactivation, UTI, and BSI. AUC values ranged from 0.63 to 0.925. Nine studies reported an AUC above 0.80, indicating good discriminative ability. The BSI model exhibited the highest AUC (0.925) (7), followed by the early post-transplant infection model (AUC = 0.914) (14) and the overall infection model (AUC = 0.900) (15). These findings suggest that the models may effectively identify high-risk individuals within their own study populations. However, PROBAST assessment revealed that all 15 studies were at a high overall risk of bias. This finding aligns with other systematic reviews of prediction models in transplantation. Haller et al. (35) reviewed 68 prediction models in living-donor transplantation and found that 94% of newly developed models and 89% of external validation studies had a high risk of bias from methodological deficiencies, with generally poor reporting quality. All 15 studies in this review used a retrospective design, drawing data from electronic medical records or clinical databases. Retrospective designs carry inherent risks of selection and recall bias, which can lead to information bias and weaken the credibility of predictor-outcome associations (18). Moreover, retrospective designs limit control for confounding variables, so their findings can only suggest associations, not confirm causation. Future studies should therefore prioritize prospective cohort or nested case-control designs to minimize information bias in model development. Blinding prevents systematic deviation in results that arises from study personnel’s knowledge or behavior. In prediction model research, blinding primarily prevents bias from information exchange between predictor assessment and outcome determination (18). 14 studies did not report blinding during predictor assessment and outcome determination. If outcome assessors know participants’ predictor information, their outcome judgment may be biased, potentially altering outcome classification. A study of 152 machine learning models found that information on outcome blinding was missing in 40% of studies and on predictor blinding in 52% (36). Lack of blinding impairs objective evaluation of subjective analytic decisions, such as variable selection and model selection. These gaps could lead to detection and performance bias, and may even overestimate effect sizes. Future studies should establish an independent data collection team and an outcome adjudication committee, with the former blinded to outcomes and the latter blinded to predictors.

All 15 studies were rated high risk in the analysis domain, the primary driver of their overall high risk of bias. Specific issues are as follows. (1) Insufficient sample size and low EPV. PROBAST guidelines recommend ensuring an EPV of at least 20 to control overfitting risk during model development (18). Insufficient sample size is a primary driver of overfitting in prediction models. Riley et al. (37) stated in their BMJ guidance that prediction models need adequate sample sizes to minimize overfitting and maintain the shrinkage factor near 1. Low EPV leads to optimism bias, whereby the model spuriously exhibits good performance in the development dataset (38). (2) Inappropriate variable selection methods. Selecting predictors based on univariable *P*-values is a common but methodologically flawed strategy in prediction model development. This strategy ignores potential interactions and collinearity among variables, thereby increasing overfitting risk. In contrast, six studies adopted shrinkage methods or machine learning approaches, including LASSO regression and recursive feature elimination. These methods constrain model parameters via a penalty term, markedly reducing overfitting risk. When the number of variables exceeds the sample size, traditional methods may fail, while regularization methods can shrink irrelevant variable coefficients to zero via an L1 penalty, achieving dimensionality reduction (39). (3) Opaque missing data handling. Liu et al. (40) noted that missing data are a common challenge in prediction model development. In 46.7% of our study, incomplete cases were directly excluded. Complete-case analysis excludes incomplete cases, introducing selection bias as the remaining sample may no longer represent the target population. It also reduces effective sample size, yielding imprecise estimates with wider confidence intervals (41). In accordance with established guidelines (18), we recommend that future studies clearly report the proportion of missing data for each predictor and outcome variable, specify the assumed missing data mechanism, and justify the chosen imputation method. Multiple imputation is the most recommended method for missing data handling. It generates multiple complete datasets, imputes each, and combines the results, effectively handling data missing at random (42). Digitale et al. (43) compared several missing data handling methods and found that traditional multiple imputation approaches, developed for inferential statistics, may not be optimal for prediction models. Imputation method performance varied more for binary than continuous outcomes, and missing data amount influenced performance more than the missing mechanism. Thus, researchers should consider the model’s specific objective and outcome type when selecting an imputation strategy. Sensitivity analyses should then be performed to compare the results of complete-case analysis with those obtained from imputation, in order to assess the robustness of the findings. Finally, imputation details should be reported transparently, including the number of imputations, the imputation model used, and the method for combining imputed values. (4) Incomplete model performance evaluation. A complete performance evaluation should cover at least three core dimensions including discrimination, calibration, and clinical utility. Although all included studies reported discrimination, three did not report calibration methods, and most did not report clinical utility. Calibration and discrimination are independent but equally important dimensions in clinical prediction model evaluation. Discrimination measures the model’s ability to correctly rank individuals with versus without the outcome, usually expressed as the C-statistic or AUC. Calibration measures agreement between predicted probabilities and observed outcome frequencies. A complete calibration assessment should include at least a calibration plot, along with quantitative reporting of the calibration intercept and slope (44). Studies show that only 32% of clinical prediction models undergo calibration assessment during development and internal validation–meaning nearly 70% of development studies overlook this critical dimension (45). Failure to report calibration metrics precludes quantifying the direction and extent of miscalibration, and prevents identifying the specific type and degree of poor calibration. Furthermore, when one model has better discrimination and another better calibration, decision curve analysis (DCA) should be used for evaluation. DCA quantifies net benefit across different threshold probabilities, thereby providing a clear basis for model comparison (46). Without DCA reporting, no criteria exist to judge a model’s value at various risk thresholds, making it difficult for decision-makers to assess its clinical merit (47). (5) Inadequate model validation. Internal validation assesses whether a model is overfitted during development (48). Although all studies performed internal validation, most did not conduct external validation. External validation is indispensable before applying a prediction model in clinical practice. Without external validation, researchers cannot determine whether model performance remains stable across different regions or healthcare settings. Such models may perform well only in the development dataset sample, and their discrimination and calibration may deteriorate markedly when applied to broader populations.

### 4.4 Predictive factors of the model

The included models contained 3–18 predictors. The most common predictors across models were age, albumin, diabetes or glycemia, kidney function indicators, and donor source. Age is a fundamental indicator of infection risk in kidney transplant recipients. Schröter et al. (49) analyzed the German Center for Infection Research Transplant Cohort and found that older recipients had significantly higher unadjusted 5-years incidences of graft failure, death, and infection than younger ones. Pneumonia, UTI, and invasive opportunistic infections all increased markedly with age. Fayos et al. (50) similarly reported that infection after kidney transplantation risk increases with age, especially in recipients over 60 years. This age-related increase reflects immunosenescence. Thymic involution reduces naive T-cell production and T-cell receptor repertoire diversity (51). In parallel, older patients have weakened ciliary clearance and diminished cough reflex sensitivity, increasing infection susceptibility (52). Albumin was the most frequently identified predictor, appearing across all infection types except UTI. Pretransplant hypoalbuminemia is associated with elevated risks of BK virus and, to a lesser extent, CMV infection after kidney transplantation. Hypoalbuminemia may thus serve as a biomarker for latent virus reactivation (53). Albumin functions to bind endotoxins and protect the endothelial glycocalyx, and a low albumin level reflects a malnourished state. Moreover, hypoalbuminemia can suppress innate immune responses by promoting granuloma formation and reducing collagen synthesis (54). Diabetes or preoperative hyperglycemia was a shared predictor for UTI (19, 22), pulmonary infection (6, 21), BSI (7), and overall infection (15). A retrospective study of 295 recipients observed markedly higher infection susceptibility in patients with diabetes (55). Notably, Yao et al. (6) reported that elevated preoperative blood glucose, rather than a history of diabetes itself, was an independent risk factor for pulmonary infection. This finding suggests that postoperative glucose management is equally crucial, even without a diabetes history. Hyperglycemia can activate NADPH oxidase, leading to elevated reactive oxygen species (ROS) levels (56). Excess ROS induces aberrant accumulation of neutrophil extracellular traps (NETosis). Excessive or dysregulated NETosis releases abundant intracellular contents, causing tissue damage and persistently amplifying inflammation, thereby aggravating lung injury (57). Kidney function indicators are also important predictors of infection after kidney transplantation. A decline in estimated glomerular filtration rate (eGFR) reflects impaired graft function and is associated with immunosuppressant accumulation and increased infection risk (58). Proteinuria suggests potential structural or functional damage in the transplanted kidney. Proteinuria often accompanies tubular dysfunction. Impaired tubular function weakens local immune defense mechanisms including antimicrobial peptide production and urine flow maintenance, thereby increasing the risk of pathogen colonization and ascending infection (59). Donor source also predicts infection after kidney transplantation. Several studies have confirmed that deceased donor recipients have a higher infection after kidney transplantation risk than living donor recipients (33, 60, 61). Some deceased donors have underlying diseases or latent infections, and donor pathogens can be directly transmitted to the recipient during transplantation (62). Moreover, deceased donor recipients typically receive more intensive immunosuppressive induction therapy to prevent acute rejection, which raises their risk of opportunistic infections. Cold ischemia time is substantially longer for deceased than for living donor kidneys (63). Prolonged cold ischemia time induces ischemia-reperfusion injury (IRI) (64). IRI promotes pro-inflammatory cytokine release via NF-κB pathway activation, leading to sterile inflammation, and simultaneously impairs dendritic cell and macrophage immune functions (65, 66).

Yao et al. (6) incorporated positive psychological capital and the Insomnia Severity Index into a prediction model for the first time. Kidney transplant recipients face multiple chronic psychological stressors, and 37% experience persistent sleep disturbances post-transplant (67). Chronic psychological stress elevates glucocorticoid levels via the hypothalamic-pituitary-adrenal axis and the sympatho-adreno-medullary system, thereby suppressing cellular immune function (68). Chronic sleep deprivation induces a systemic low-grade inflammatory state, characterized by elevated pro-inflammatory cytokines such as IL-1β and TNF-α (69). Future studies could therefore consider incorporating psychological factors into prediction models, weighing their clinical value. Guo et al. (20) introduced CT-based radiomics into a prediction model. Traditional clinical assessment relies on subjective physician interpretation, whereas radiomics automatically extracts hundreds of quantitative parameters from CT images via high-throughput computation. These parameters reflect microscopic tissue heterogeneity and subclinical lung pathology (70), offering an objective basis for detecting early infection signs that immunosuppression may mask. The platelet to lymphocyte (PLR) is a significant risk factor for infection after kidney transplantation and is strongly associated with long-term infectious events and chronic renal dysfunction in kidney transplant recipients (71). Platelets act as immunomodulatory cells. Upon activation, they release pro-inflammatory mediators such as CD40 ligand (CD40L) and P-selectin and participate in both innate and adaptive immunity (72, 73). After kidney transplantation, in areas of IRI in the graft, complement activation induces platelet aggregation in damaged microvessels. Platelets then cooperate with neutrophils to release reactive oxygen species and proteolytic enzymes, aggravating tissue injury and creating conditions favorable for secondary infection (74). In a retrospective cohort study of 1,512 deceased donor recipients, delayed graft function (DGF) was independently associated with increased UTI and BK viremia risk within the first post-transplant year (75). The core pathological basis of DGF is severe IRI. During cold preservation, ATP depletion leads to mitochondrial dysfunction and cellular edema. After reperfusion, a reactive oxygen species burst occurs, and injured tubular and endothelial cells release damage-associated molecular patterns (DAMPs), including high mobility group box 1 (HMGB1), heat shock proteins, and mitochondrial DNA (76). These DAMPs activate the NLRP3 inflammasome pathway via Toll-like receptors 2 and 4 (TLR2/4), driving neutrophils and macrophages to release inflammatory cytokines and thereby generating a robust, non-infectious inflammatory microenvironment within the graft (77). This pro-inflammatory state depletes innate immunity’s bactericidal reserve and impairs systemic immune surveillance by inducing dendritic cell maturation defects (76, 78). Furthermore, during the first week after kidney transplantation, DGF patients often require a central venous catheter for dialysis. The catheter breaches the skin barrier and provides a portal of entry for *Staphylococcus epidermidis*, *Staphylococcus aureus*, and *Enterococcus* species into the bloodstream (79). Biofilms on the catheter surface can release planktonic bacteria, serving as a persistent bacteremia source (80). The prolonged hospital stay resulting from delayed kidney function recovery also increases the risk of colonization with multidrug-resistant nosocomial pathogens, such as methicillin-resistant *Staphylococcus aureus* (MRSA), vancomycin-resistant *Enterococcus* (VRE), and extended-spectrum beta-lactamase (ESBL)-producing Enterobacteriaceae (81). Acute rejection and induction therapy are also potential predictors. Acute T cell-mediated rejection (TCMR) is the most common acute rejection type after kidney transplantation. Its pathogenesis involves recipient CD4^+^ and CD8^+^ T cells recognizing donor HLA antigens, becoming activated, and mediating graft injury through direct cytotoxicity and inflammatory cytokine release (82). Methylprednisolone pulse therapy, a first-line treatment, inhibits pro-inflammatory transcription factors such as NF-κB and suppresses genes involved in T-cell activation. It also reduces neutrophil and macrophage chemotaxis and phagocytic function, thereby increasing susceptibility to bacterial and fungal infections (83). Different induction regimens affect infection after kidney transplantation risk differently. ATG depletes peripheral circulating T cells via complement-dependent lysis and apoptosis, leading to a sharp, sustained decline in CD4^+^ and CD8^+^ T-cell counts (84). In a retrospective study of 500 recipients, Lee et al. reported that ATG treatment in the acute rejection subgroup independently predicted *Pneumocystis jirovecii* pneumonia (PJP). This finding suggests that severe T-cell depletion may create conditions favorable for opportunistic fungal infections (85).

### 4.5 Implications and limitations

This study provides the first systematic review of existing prediction models for infection after kidney transplantation. It may help clinicians compare the predictive performance, target population characteristics, and clinical accessibility of predictors, thereby guiding selection of the most appropriate tool for a given scenario. Research on prediction models for infection after kidney transplantation started relatively late and remains exploratory. Although models based on multidimensional parameters such as immune status and clinical characteristics have been developed, no prediction model for infection after kidney transplantation has yet been recommended by international guidelines for routine clinical use. Haller et al. (35) cautioned against using prediction models that are poorly developed, inadequately reported, or insufficiently validated. Clinicians should interpret existing model predictions cautiously. These models should serve as risk stratification tools to support clinical judgment, not as a definitive basis for decisions. Before implementation, healthcare institutions should validate a model using local data to assess its reliability and applicability, thereby avoiding misjudgments from indiscriminate adoption of external models. Moreover, most models are limited to single-center retrospective studies and lack external validation, so their generalizability requires further confirmation. Future studies should adopt prospective multicenter designs, use sound methods for variable selection and missing data handling, strengthen external validation and calibration assessment, and explore dynamic prediction models and machine learning algorithms. Furthermore, future research should strictly adhere to PROBAST and Transparent reporting of a multivariable prediction model for individual prognosis or diagnosis (TRIPOD) reporting standards to promote substantive progress toward clinical translation of after kidney transplantation prediction models.

This review systematically examined predictor characteristics in the included models. The predictor spectrum varied across infection outcomes. Thus, predictor selection should not mechanically apply a uniform set of variables across different infections. Instead, it should be tailored to the target infection’s pathogenic mechanisms. For example, existing models for UTI generally lack assessment of preoperative urinary tract anatomy and function. Researchers are advised to incorporate urinary system-specific variables, such as a history of recurrent UTI and urine flow rate, into the candidate predictor pool. BK virus activation models have overlooked dynamic lymphocyte subset trends and immune function, and lack information on donor-recipient BK virus neutralizing antibody serotype mismatch. Future studies should include dynamic lymphocyte subsets, donor/recipient BK virus serostatus, and eGFR slope as core candidate predictors. Methodologically, priority should be given to dynamic Cox regression or landmark models to capture the time-varying effects of predictors. For BSI prediction models, future studies should accurately record central venous catheter type, catheterization duration, and maintenance practices, and should establish an attributable window from catheter exposure to BSI onset. Preoperative gut microbiota composition, intestinal barrier function markers, or neutrophil-to-lymphocyte dynamics could also be explored. Models using overall infection as a composite outcome have broad clinical applicability, but the pathogenic mechanisms of different infection types are highly heterogeneous. Combining multiple infections into a single outcome may dilute type-specific predictor effects. Therefore, overall infection models should serve as a primary screening tool, not a decision basis for precision prevention. In terms of predictor selection, preference should be given to integrating indicators of shared pathways across infection types, such as kidney function, immunosenescence, and graft-related factors. Overall infection models and pathogen-specific tools serve fundamentally distinct purposes in clinical practice. Overall infection models are designed to capture the net effect of multiple risk pathways, including the overall state of immunosuppression, baseline patient characteristics, and graft function, all of which predispose recipients to a broad spectrum of infectious complications (86). These models are inherently non-specific. In contrast, pathogen- or site-specific tools are constructed around variables that are mechanistically linked to particular infectious entities. In clinical practice, we recommend a sequential approach. Clinicians should first apply an overall infection model for broad risk stratification to identify patients at elevated baseline risk. For patients identified as high-risk by these models, or for those presenting with specific clinical scenarios, pathogen- or site-specific models should then be applied to further refine risk assessment for particular infection types. The output of this second step can inform targeted preventive strategies, such as extending antimicrobial prophylaxis, intensifying viral load monitoring, or adjusting immunosuppression regimens (86).

This systematic review has several limitations. First, only studies published in English and Chinese were included, which may introduce language bias. Second, as PROBAST revealed, methodological shortcomings in the original studies further limited our analysis depth and may have introduced additional bias. Third, substantial heterogeneity among the included studies precluded quantitative synthesis, so the analysis was limited to qualitative description.

## 5 Conclusion

In summary, this study systematically reviewed the prediction models for infection after kidney transplantation. A total of 15 studies were included, covering multiple outcome types such as pulmonary infection, overall infection, BK virus activation, UTI, and BSI. Although existing models showed apparently good discriminative ability in internal validation, this finding must be interpreted with caution due to the uniformly high risk of bias and the well-recognized phenomenon of optimism bias, whereby high internal AUCs may not reflect true predictive performance. Moreover, these models also suffered from substantial methodological limitations that limit their clinical applicability. Future efforts should therefore focus on further optimization and external validation of existing models, so as to facilitate the rapid and accurate identification of high-risk individuals for after kidney transplantation in clinical settings.

## Data Availability

The original contributions presented in this study are included in this article/Supplementary material, further inquiries can be directed to the corresponding author.
