# Supplementary material for: Prediction models for infection after kidney transplantation: a systematic review
Source: Front Med (Lausanne). 2026 Sep 10;13:1916764. doi: 10.3389/fmed.2026.1916764 (PMC13600904; doi:10.3389/fmed.2026.1916764)
Supplement: Supplementary file 1 [file Data_Sheet_1.pdf]

Supplementary Material

1 Supplementary Data

Table 1 PICOTS framework

Framework dimension	Definition and content
P (Population)	Kidney transplant recipients
I (Index)	Multivariable risk prediction models
C (Comparator)	None
O (Outcome)	Postoperative infections including but not limited to:<br>1.Specific pathogens: BK virus reactivation, CMV.<br>2.Infection sites: pulmonary infection, UTI, BSI.<br>3.Overall infection: any type of infection occurring after transplantation.
T (Timing)	Specific postoperative time windows:<br>1.Within 1 year post- transplant<br>2.Within 30 days post- transplant<br>3.During hospitalization post- transplant<br>4.Within 6 months post- transplant.
S (Setting)	Clinical healthcare settings

CMV: cytomegalovirus; UTI: urinary tract infection; BSI: bloodstream infection

Table 2 Search strategy

CNKI		
26/3/2026		
#1	SU=肾移植+肾脏移植+肾移植术<br><br>***Translation Content:*** *SU=肾移植 (kidney transplantation) + 肾脏移植 (renal transplantation) + 肾移植 (kidney transplant)*	25,300
#2	SU=感染+细菌感染+病毒感染+真菌感染+脓毒症+肺炎+尿路感染+巨细胞病毒+BK 病毒<br><br>***Translation Content:*** *SU=感染 (infection) + 细菌感染 (bacterial infection) + 病毒感染 (viral infection) + 真菌感染 (fungal infection) + 脓毒症 (sepsis) + 肺炎 (pneumonia) + 尿路感染 (urinary tract infection) + 巨细胞病毒 (cytomegalovirus, CMV) + BK 病毒 (BK virus)*	1,871,598
#3	SU=风险分层+风险评分+风险评估+风险预测+机器学习+列线图+模型+诺莫图+算法+危险分层+影响因素+危险因素+影响因子+预测+预测工具+预测模型+预测因子+诊断模型<br><br>***Translation Content:*** *SU=风险分层 (risk stratification) + 风险评分 (risk score) + 风险评估 (risk assessment) + 风险预测 (risk prediction) + 机器学习 (machine learning) + 列线图 (nomogram) + 模型 (model) + 诺莫图 (nomogram) + 算法 (algorithm) + 危险分层 (risk stratification) + 影响因素 (influencing factors) + 危险因素 (risk factors) + 影响因子 (influencing factors) + 预测 (prediction) + 预测工具 (prediction tool) + 预测模型 (prediction model) + 预测因子 (predictor) + 诊断模型 (diagnostic model)*	7,696,100
#4	#1 AND #2 AND #3	390
Wanfang Database		
26/3/2026		
#1	题名或关键词:(肾移植 or 肾脏移植 or 肾移植术)<br><br>***Translation Content:*** *TI or KY:(肾移植 (kidney transplantation) or 肾脏移植 (renal transplantation) or 肾移植术 (kidney transplant surgery))*	58,862
#2	题名或关键词:(感染 or 细菌感染 or 病毒感染 or 真菌感染 or 脓毒症 or 肺炎 or 尿路感染 or 巨细胞病毒 or BK 病毒)<br><br>***Translation Content:*** *TI or KY: (感染 (infection) or 细菌感染 (bacterial infection) or 病毒感染 (viral infection) or 真菌感染 (fungal infection) or 脓毒症 (sepsis) or 肺炎 (pneumonia) or 尿路感染 (urinary tract infection) or 巨细胞病毒 (cytomegalovirus, CMV) or BK 病毒 (BK virus))*	164,777
#3	题名或关键词:(风险分层 or 风险评分 or 风险评估 or 风险预测 or 机器学习 or 列线图 or 模型 or 诺莫图 or 算法 or 危险分层 or 影响因素 or 危险因素 or 影响因子 or 预测 or 预测工具 or 预测模型 or 预测因子 or 诊断模型)<br><br>***Translation Content:*** *TI or KY:(风险分层 (risk stratification) or 风险评分 (risk score) or 风险评估 (risk assessment) or 风险预测 (risk prediction) or 机器学习 (machine learning) or 列线图 (nomogram) or 模型 (model) or 诺莫图 (nomogram) or 算法 (algorithm) or 危险分层 (risk stratification) or 影响因素 (influencing factors) or 危险因素 (risk factors) or 影响因子 (influencing factors) or 预测 (prediction) or 预测工具 (prediction tool) or 预测模型 (prediction model) or 预测因子 (predictor) or 诊断模型 (diagnostic model))*	8,012,292
#4	#1 AND #2 AND #3	138
China Biomedical Literature Database (SinoMed)		
26/3/2026		

#1	"肾移植"[加权:扩展] OR "肾脏移植"[摘要:智能] OR "肾移植术"[摘要:智能]<br><br>***Translation Content:*** *"肾移植 (kidney transplantation)" [加权:扩展 (weighted: expanded)] OR "肾脏移植 (renal transplantation)" [摘要:智能 (abstract: intelligent)] OR "肾移植术 (kidney transplant surgery)" [摘要:智能 (abstract: intelligent)]*	22280
#2	"感染"[加权:扩展] OR("感染"[摘要:智能] OR "细菌感染"[摘要:智能] OR "病毒感染"[摘要:智能] OR "真菌感染"[摘要:智能] OR "脓毒症"[摘要:智能] OR "肺炎"[摘要:智能] OR "尿路感染"[摘要:智能] OR "巨细胞病毒"[摘要:智能] OR "BK 病毒"[摘要:智能])<br><br>***Translation Content:*** *"感染 (infection)" [加权:扩展 (weighted: expanded)] OR ("感染 (infection)" [摘要:智能 (abstract: intelligent)] OR "细菌感染 (bacterial infection)" [摘要:智能 (abstract: intelligent)] OR "病毒感染 (viral infection)" [摘要:智能 (abstract: intelligent)] OR "真菌感染 (fungal infection)" [摘要:智能 (abstract: intelligent)] OR "脓毒症 (sepsis)" [摘要:智能 (abstract: intelligent)] OR "肺炎 (pneumonia)" [摘要:智能 (abstract: intelligent)] OR "尿路感染 (urinary tract infection)" [摘要:智能 (abstract: intelligent)] OR "巨细胞病毒 (cytomegalovirus, CMV)" [摘要:智能 (abstract: intelligent)] OR "BK 病毒 (BK virus)" [摘要:智能 (abstract: intelligent)])*	1,763,567
#3	("风险分层"[摘要:智能] OR "风险评分"[摘要:智能] OR "风险评估"[摘要:智能] OR "风险预测"[摘要:智能] OR "机器学习"[摘要:智能] OR "列线图"[摘要:智能] OR "模型"[摘要:智能] OR "诺莫图"[摘要:智能] OR "算法"[摘要:智能] OR "危险分层"[摘要:智能] OR "影响因素 OR 危险因素"[摘要:智能] OR "影响因子"[摘要:智能] OR "预测"[摘要:智能] OR "预测工具"[摘要:智能] OR "预测模型"[摘要:智能] OR "预测因子"[摘要:智能] OR "诊断模型"[摘要:智能])<br><br>***Translation Content:*** *("风险分层 (risk stratification)" [摘要:智能 (abstract: intelligent)] OR "风险评分 (risk score)" [摘要:智能 (abstract: intelligent)] OR "风险评估 (risk assessment)" [摘要:智能 (abstract: intelligent)] OR "风险预测 (risk prediction)" [摘要:智能 (abstract: intelligent)] OR "机器学习 (machine learning)" [摘要:智能 (abstract: intelligent)] OR "列线图 (nomogram)" [摘要:智能 (abstract: intelligent)] OR "模型 (model)" [摘要:智能 (abstract: intelligent)] OR "诺莫图 (nomogram)" [摘要:智能 (abstract: intelligent)] OR "算法 (algorithm)" [摘要:智能 (abstract: intelligent)] OR "危险分层 (risk stratification)" [摘要:智能 (abstract: intelligent)] OR "影响因素 (influencing factors) OR 危险因素 (risk factors)" [摘要:智能 (abstract: intelligent)] OR "影响因子 (influencing factors)" [摘要:智能 (abstract: intelligent)] OR "预测 (prediction)" [摘要:智能 (abstract: intelligent)] OR "预测工具 (prediction tool)" [摘要:智能 (abstract: intelligent)] OR "预测模型 (prediction model)" [摘要:智能 (abstract: intelligent)] OR "预测因子 (predictor)" [摘要:智能 (abstract: intelligent)] OR "诊断模型 (diagnostic model)" [摘要:智能 (abstract: intelligent)])*	992,955
#4	(#1) AND (#2) AND (#3)	207
China Science and Technology Journal Database (VIP Database)		
26/3/2026		
#1	M=(肾移植 or 肾脏移植 or 肾移植术) and M=(感染 or 细菌感染 or 病毒感染 or 真菌感染 or 脓毒症 or 肺炎 or 尿路感染 or 巨细胞病毒 or BK 病毒) and M=(风险分层 or 风险评分 or 风险评估 or 风险预测 or 机器学习 or 列线图 or 模型 or 诺莫图 or 算法 or 危险分层 or 影响因素 or 危险因素 or 影响因子 or 预测 or 预测工具 or 预测模型 or 预测因子 or 诊断模型)<br><br>***Translation Content:*** *M=(肾移植 (kidney transplantation) or 肾脏移植 (renal transplantation) or 肾移植术 (kidney transplant surgery)) and M=(感染 (infection) or 细菌感染 (bacterial infection) or 病毒感染 (viral infection) or 真菌感染 (fungal infection) or 脓毒症 (sepsis) or 肺炎 (pneumonia) or 尿路感染 (urinary tract infection) or 巨细胞病毒 (cytomegalovirus, CMV) or BK 病毒 (BK virus)) and M=(风险分层 (risk stratification) or 风险评分 (risk score) or 风险评估 (risk assessment) or 风险预测 (risk prediction) or 机器学习 (machine learning) or 列线图 (nomogram) or 模型 (model) or 诺莫图 (nomogram) or 算法 (algorithm) or 危险分层 (risk stratification) or 影响因素 (influencing*	145

	factors) or 危险因素 (risk factors) or 影响因子 (influencing factors) or 预测 (prediction) or 预测工具 (prediction tool) or 预测模型 (prediction model) or 预测因子 (predictor) or 诊断模型 (diagnostic model))	
Pubmed		
26/3/2026		
#1	((Kidney Transplantation[Mesh])) OR (kidney transplant*[Title/Abstract] OR renal transplant*[Title/Abstract] OR kidney graft*[Title/Abstract] OR renal graft[Title/Abstract])	136,749
#2	("Infections"[Mesh]) OR (infection*[Title/Abstract] OR sepsis[Title/Abstract] OR septic[Title/Abstract] OR pneumonia[Title/Abstract] OR urinary tract infection[Title/Abstract] OR bacteriuria[Title/Abstract] OR cytomegalovirus[Title/Abstract] OR BK virus[Title/Abstract] OR polyomavirus[Title/Abstract] OR fungal[Title/Abstract] OR candida[Title/Abstract] OR aspergillus[Title/Abstract])	4,437,078
#3	(machine learning[Title/Abstract] OR nomogram[Title/Abstract] OR prediction model[Title/Abstract] OR prediction tool[Title/Abstract] OR prediction tool*[Title/Abstract] OR predictive model[Title/Abstract] OR predictive tool[Title/Abstract] OR predictive value[Title/Abstract] OR predictives score[Title/Abstract] OR prognostic model[Title/Abstract] OR warning model[Title/Abstract])	564,783
#4	#1 AND #2 AND #3	324
Embase		
26/3/2026		
#1	'kidney transplantation':ti,ab,kw OR 'kidney transplant*':ti,ab,kw OR 'renal transplant*':ti,ab,kw OR 'kidney graft*':ti,ab,kw OR 'renal graft':ti,ab,kw	174,157
#2	'machine learning':ti,ab,kw OR nomogram:ti,ab,kw OR 'prediction model':ti,ab,kw OR 'prediction tool':ti,ab,kw OR 'prediction tool*':ti,ab,kw OR 'predictive model':ti,ab,kw OR 'predictive tool':ti,ab,kw OR 'predictive value':ti,ab,kw OR 'predictives score':ti,ab,kw OR 'prognostic model':ti,ab,kw OR 'warning model':ti,ab,kw	548,703
#3	infections:ti,ab,kw OR infection*:ti,ab,kw OR sepsis:ti,ab,kw OR septic:ti,ab,kw OR pneumonia:ti,ab,kw OR 'urinary tract infection':ti,ab,kw OR bacteriuria:ti,ab,kw OR cytomegalovirus:ti,ab,kw OR 'bk virus':ti,ab,kw OR polyomavirus:ti,ab,kw OR fungal:ti,ab,kw OR candida:ti,ab,kw OR aspergillus:ti,ab,kw	3,286,315
#4	#1 AND #2 AND #3	413
Cochrone library		
26/3/2026		
#1	AT=Kidney Transplantation OR kidney transplant* OR renal transplant* OR kidney graft* OR renal graft	22,893
#2	AT=Infections OR infection* OR sepsis OR septic OR pneumonia OR urinary tract infection OR bacteriuria OR cytomegalovirus OR BK virus OR polyomavirus OR fungal OR candida OR aspergillus	191,209
#3	AT=machine learning OR nomogram OR prediction model OR prediction tool OR prediction tool* OR predictive model OR predictive tool OR predictive value OR predictives score OR prognostic model OR warning model	23,207
#4	#1 AND #2 AND #3	285
Web of Science		
26/3/2026		
#1	TI=(Kidney Transplantation OR kidney transplant* OR renal transplant* OR kidney graft* OR renal graft)	93,798
#2	TI=(Infections OR infection* OR sepsis OR septic OR pneumonia OR urinary tract infection OR bacteriuria OR cytomegalovirus OR BK virus OR polyomavirus OR fungal OR candida OR aspergillus)	1,456,191
#3	TI=(machine learning OR nomogram OR prediction model OR prediction tool OR prediction tool* OR predictive model OR predictive tool OR predictive value OR predictives score OR prognostic model OR warning model)	1,451,782
#4	#1 AND #2 AND #3	472

Table 3 Basic characteristics of the included studies

Study	Country	Study design	Sample size	Study population	Outcome	Donor type	Infection incidence	Timing of infection	Multi-/Single-center	Predictive factors
Chu et al., 2025(19)	China	Retrospective case-control study	77	Adult kidney transplant recipients	UTI	DBD	31/77 (40.3%)	Within 1 year post-transplant	Single-center	PCT, UA, Urea
Guo et al., 2025(20)	China	Retrospective cohort study	110	First time kidney transplant recipient	Pulmonary infection	NR	70/110 (63.6%)	Within 1 year post-transplant	Single-center	SaO2, SBP, 14 Radiomics features
Yao et al., 2025(6)	China	Retrospective cohort study	327	First time adult kidney transplant recipient	Pulmonary infection	NR	102/327 (31.2%)	Within 1 year post-transplant	Single-center	Age, Preoperative blood glucose, Postoperative day 5 white blood cell count, ISI, Positive psychological capital, Cyclosporine use, Mycophenolate mofetil use
Gong et al., 2022(21)	China	Retrospective cohort study	197	First time kidney transplant recipient	Pulmonary infection	BDB and living related donors	42/197 (21.3%)	Within 1 year post-transplant	Single-center	Smoking, Diabetes, Pulmonary disease, Albumin <35 g/L
Cui et al., 2025(7)	China	Retrospective cohort study	237	Adult recipients of allogeneic kidney transplantation	BSI	NR	26/237 (11.0%)	Within 6 months post-transplant	Single-center	Diabetes, ALB, PLT
Zhang et al., 2026(15)	China	Retrospective cohort study	268	Adult kidney transplant recipients	Overall infectious diseases	Living and deceased	96/268 (35.8%)	Within 1 year post-transplant	Single-center	Smoking, Age, Donor type, Diabetes, Operative duration, ALB
Bian et al., 2025(22)	China	Retrospective cohort study	759	Adult kidney transplant recipients	UTI	Living and deceased	200/759 (26.4%)	Within 1 year post-transplant	Single-center	Recipient Gender (female), Donor type, Diabetes, Acute rejection, Delayed catheter removal (>7 days), Stent not removed
Wang et al., 2022(23)	China	Retrospective cohort study	310	Adult kidney transplant recipients	BK virus activation	Living and deceased	67/310 (21.6%)	Detected during routine follow- up	Single-center	Donor source, Direct bilirubin, NE
Chen et al., 2022(24)	China	Retrospective cohort study	863	Adult kidney transplant recipients	Overall infectious diseases	NR	407/863 (47.2%)	Median 62.3 days (IQR 46.9–90.6) post-discharge	Single-center	Albumin infusion requirement, Red blood cell infusion requirement, TG, UA, Cr, Globulin, Neutrophil percentage, WBC
Fernández - Ruiz et al.,	Spain	Retrospective cohort study	932	Adult kidney transplant recipients	Overall infectious diseases	DBD and DCD	238/932 (25.5%)	Between post-transplant months 1 and 6	Multicenter	Recipient age ≥62 years, eGFR <37 ml/min at month 1, Infection within first month,

2020(25)										CD4+ count <40 cells/μl, CD8+ count <155 cells/μl, IgG <500 mg/dl, C3 <78 mg/dl
Pan et al., 2026(13)	China	Retrospective cohort study	865	Adult kidney transplant recipients	Pulmonary infection	Living and deceased	186/865 (21.5%)	Postoperative period	Single-center	Recipient Gender（male）, History of multiple transplants, Hypertension, Cardiac disease, Postoperative rejection
Yamauchi et al., 2025(5)	USA	Retrospective cohort study	560	Adult kidney transplant recipients	BKPyVAN	Living and deceased	75/560 (13.4%)	Within 1 year post-transplant	Single-center	Age>50 years, Recipient Gender（male）, Prior kidney transplant
Fang et al., 2022(26)	China	Retrospective cohort study	200	Adult kidney transplant recipients	BK virus reactivation	NR	121/200 (60.5%)	Within 2.5–8.5 months post-transplant	Single-center	Recipient Gender, Age, BMI, Induction regimen, DGF, AR, Estimated eGFR, uPRO, uWBC, Urinary erythrocyte, Serum albumin, PLT, Tacrolimus concentration, NE, Blood lymphocyte count, UA, CMV infection, Dialysis time
Luo et al., 2020(27)	China	Retrospective cohort study	519	Non pediatric kidney transplant recipients	Severe pneumonia	Deceased donor only	43/519 (8.3%)	During post-transplant hospitalization	Single-center	Preoperative pulmonary lesions, Reoperation, Recipient age, Preoperative immunoglobulin level, DGF, rATG, Preoperative albumin, Donor age
Xu et al., 2025(14)	China	Retrospective cohort study	410	Adult kidney transplant recipients	Overall early postoperative infections	Living and deceased	131/410 (32.0%)	Within 30 days post-transplant	Single-center	Smoking, Drainage tube indwelling time, Catheter indwelling time, ALB, Hb

AR: Acute rejection; ALB: Albumin; BSI: Bloodstream infection; SaO2: Arterial oxygen saturation; BMI: Body mass index; BKPyVAN: BK polyomavirus-associated nephropathy; Cr: Creatinine; CMV infection: Cytomegalovirus infection; DBD: Donation after Brain Death; DCD: donation after circulatory death; DGF: Delayed graft function; eGFR: Estimated glomerular filtration rate; Hb: Hemoglobin; H-L test: Hosmer-Lemeshow; ISI: Insomnia Severity Index; LR:Logistic regression; NE: neutrophil count; NR: Not Reported; PLT: Platelet count; PCT: Procalcitonin; rATG dose: Rabbit anti-human thymocyte globulin dose; SBP: Systolic blood pressure; TG: Triglyceride; UA: Uric acid; uRBC: Urinary erythrocyte; uPRO: Urinary protein; UTI: Urinary tract infection; WBC: White blood cell count;

Table 4 Characteristics of the prediction models in the included studies

Study	Variable selection	Model	Model verification method	Handling of continuous variables	Handling of missing data	Calibration method	Model presentation
Chu et al., 2025(19)	Univariate + multivariate logistic regression	LR	Random split Cross-Validation	Continuous	Excluded	Calibration curve	Nomogram
Guo et al., 2025(20)	Univariate + multivariate logistic regression (clinical); LASSO + 10-fold CV (radiomics)	LR	Random split Cross-Validation	Continuous	Excluded	H-L test	NR
Yao et al., 2025(6)	Univariate + multivariate logistic regression	LR	Cross-Validation	Continuous	Excluded	NR	Logistic regression equation
Gong et al., 2022(21)	Univariate + multivariate logistic regression	LR	External validation	Classified	Excluded	H-L test	Logistic regression equation
Cui et al., 2025(7)	LASSO + multivariate logistic regression	LR	Bootstrap Cross-Validation	Continuous	Multiple imputation for missing <5% random; excluded if high missing	H-L test, Calibration curve	Nomogram
Zhang et al., 2026(15)	Univariate + LASSO + multivariate logistic regression	LR	Bootstrap Random split	Continuous	NR	H-L test, Calibration curve	Nomogram
Bian et al., 2025(22)	Univariate + multivariate logistic regression	LR	Random split	Continuous	Excluded if missing >10%; multiple imputation for <10%	H-L test, Calibration curve	Nomogram
Wang et al., 2022(23)	Univariate + multivariate logistic regression	LR	External validation	Continuous	NR	H-L test	Nomogram
Chen et al., 2022(24)	LASSO regression	LR	Random split	Continuous	NR	Calibration curve	Nomogram
Fernández-Ruiz et al., 2020(25)	Univariate + multivariate logistic regression	LR	Bootstrap External validation	Classified	Multiple imputation in sensitivity analysis	H-L test, Calibration curve	Integer risk score
Pan et al., 2026(13)	Univariate + multivariate logistic regression	LR	Bootstrap	Classified	Excluded	H-L test, Calibration curve	Nomogram
Yamauchi et al.,	Univariate + multivariate logistic regression	LR	Random split Cross-Validation	Classified	NR	Calibration curve	Integer risk score

2025(5)							
Fang et al., 2022(26)	Univariable time-dependent Cox (p<0.1) + backward AIC	Dynamic Cox regression, Static Cox regression	Cross-Validation	Continuous	NR	NR	Dynamic Cox regression equation
Luo et al., 2020(27)	RFECV	LR, SVM, Random Forest, Naive Bayes, AdaBoost	Random split Cross-Validation	Continuous	Excluded	NR	NR
Xu et al., 2025(14)	Univariate + multivariate logistic regression	LR	Random split	Continuous	Excluded	H-L test, Calibration curve	Nomogram

H-L test: Hosmer-Lemeshow; LR: Logistic regression; NR: Not Reported; RFECV: Recursive feature elimination with cross-validation; LASSO: Least absolute shrinkage and selection operator
